# Is There a Role for [^18^F]FDG PET-CT in Staging MALT Lymphoma?

**DOI:** 10.3390/cancers14030750

**Published:** 2022-01-31

**Authors:** Dan Cohen, Chava Perry, Shir Hazut-Krauthammer, Mikhail Kesler, Yair Herishanu, Efrat Luttwak, Einat Even-Sapir, Irit Avivi

**Affiliations:** 1 Department of Nuclear Medicine, Tel-Aviv Sourasky Medical Center, 6 Weizmann St., Tel Aviv 6423906, Israel; danco@tlvmc.gov.il (D.C.); shirkr@tlvmc.gov.il (S.H.-K.); mikhailk@tlvmc.gov.il (M.K.); evensap@tlvmc.gov.il (E.E.-S.); 2 Institute of Hematology, Tel-Aviv Sourasky Medical Center, 6 Weizmann St., Tel Aviv 6423906, Israel; chavap@tlvmc.gov.il (C.P.); yairh@tlvmc.gov.il (Y.H.); efratlu@tlvmc.gov.il (E.L.); 3 Sackler Faculty of Medicine, Tel Aviv University, Tel Aviv 6997801, Israel

**Keywords:** MALT lymphoma, [^18^F]FDG, detection rate, physiologic [^18^F]FDG-uptake, SUVmax

## Abstract

**Simple Summary:**

MALT lymphoma represents a relatively rare lymphoma subtype that arises in different extranodal anatomic locations. In the current paper we summarize our experience in assessing disease extent and metabolic characteristics of MALT lymphomas with [^18^F]FDG PET-CT at staging, and aim to highlight the strengths and challenges this imaging modality poses. In our data, all extranodal lesions located in subcutaneous-tissue, lung and liver were detected on PET, while some of those located in other tissues (such as along the digestive tract) were not detected on PET. We also evaluated the predictive role of PET in these patients, and found that increased [^18^F]FDG-uptake in extranodal lesions was associated with worse disease progression.

**Abstract:**

The role of ^18^F-fluorodeoxyglucose ([^18^F]FDG) positron emission tomography—computed tomography (PET-CT) in assessing mucosa-associated lymphoid tissue (MALT) lymphoma is debatable. We retrospectively explored the role of [^18^F]FDG PET-CT in staging and predicting progression-free-survival (PFS) of patients with newly-diagnosed MALT lymphoma. Sixty-six studies were included. The maximum standardized uptake value (SUVmax), metabolic tumor volume (MTV) and total lesion glycolysis (TLG) were documented in the “hottest” extranodal and nodal lesions. Extranodal lesions and accompanying nodal disease were detected on PET in 38/66 (57.6%) and 13/66 (19.7%) studies, respectively. Detection rate of extranodal lesions differed significantly between those located in tissues with high/heterogeneous (e.g., stomach) vs low/homogenous (e.g., subcutaneous-tissue, lung) physiologic [^18^F]FDG-uptake (40.4% vs. 100%, *p* < 0.01). Nodal lesions had significantly lower SUVmax, MTV and TLG compared with extrandodal lesions in the same patients. Detection and [^18^F]FDG-avidity of extranodal lesions were higher in patients with advanced, bulky disease and concomitant marrow/nodal involvement. Increased SUVmax of extranodal lesions predicted shorter PFS (HR 1.10, 95% CI 1.01–1.19, *p* = 0.02). Higher SUVmax and TLG showed trends towards shorter PFS in patients with localized disease. In conclusion, detection rate of extranodal MALT lymphoma lesions located in tissues with low/homogeneous physiologic [^18^F]FDG-uptake is excellent on [^18^F]FDG PET-CT. When detected, SUVmax of extranodal lesions may predict PFS.

## 1. Introduction

Mucosa-associated lymphoid tissue (MALT) lymphoma, also known as extranodal marginal zone lymphoma (MZL), involves the mucosa-associated lymphoid tissue and can potentially develop at any mucosal site, most frequently in the stomach, but also in the orbit, lung and other tissues [1,2].

MALT lymphomas usually follow an indolent clinical course and patients diagnosed with MALT lymphoma usually have a favorable prognosis. However, due to the large variability in clinical presentation, management of this heterogeneous group of patients is often patient-tailored [3,4]. At staging, patients presenting with localized disease are usually considered for radiotherapy, whereas patients with multiple extranodal lesions or concomitant nodal involvement are referred to watch-and-wait (WW) policy or systemic therapy composed of anti-CD20 antibodies with or without chemotherapy, depending on their symptoms and tumor burden. Accurate anatomical staging is therefore crucial for treatment planning.

While ^18^F-fluorodeoxyglucose ([^18^F]FDG) positron emission tomography—computed tomography (PET-CT) is the preferred imaging modality for staging most lymphoma subtypes [5], it is not considered mandatory for staging MALT lymphomas and other subtypes of MZL [6,7].

Several studies with different methodologies have evaluated the role of [^18^F]FDG PET-CT in patients with MALT lymphoma. A wide range of reported detection rates and [^18^F]FDG-avidity have led to a continuous debate as to whether MALT lymphoma should be categorized as [^18^F]FDG-avid [6,7,8,9,10,11,12,13,14,15,16,17,18,19]. There are only anecdotal conflicting reports regarding the predictive role of [^18^F]FDG PET-CT in patients with MALT lymphoma [19,20,21,22,23,24,25], and a recent meta-analysis has stressed the necessity of additional studies to confirm or contradict existing results [6].

As the role of [^18^F]FDG PET-CT in MALT lymphoma continues to be a subject of debate, in the current study we aimed to summarize our experience in this topic, and particularly use a retrospective cohort of patients to describe and explore the role of semiquantitative PET parameters in staging studies of patients with MALT lymphoma.

## 2. Materials and Methods

### 2.1. Patient Population

We retrospectively screened the medical records of all patients that met the following criteria: (i) age ≥ 18 years (ii) diagnosed with histologically proven MALT lymphoma between April 2010 and August 2021 (diagnosis confirmed by hematopathology expert) (iii) clinically evaluated and treated at the hematology institute at Tel-Aviv Soursaky Medical Center (iv) underwent a whole-body [^18^F]FDG PET-CT in the nuclear medicine department at Tel-Aviv Sourasky Medical Center (GE Healthcare; DISCOVERY 690 or DISCOVERY MI only) for primary staging.

Sixty-six patients fulfilled the inclusion criteria. For all patients, we documented the disease stage, location of the extranodal involved site, coexistent bone marrow (BM) or nodal involvement, laboratory tests results (presence of anemia, thrombocytopenia, higher LDH), pathological data (presence of high Ki-67 mitotic index, defined as above 20%) and the primary clinical strategy used immediately after staging (WW strategy, radiotherapy, systemic immune/chemoimmunotherapy, or surgical resection). Staging of MALT lymphoma involving the gastrointestinal tract was conducted according to the Lugano score for gastrointestinal lymphoma [5]. Patients with localized orbital MALT lymphoma (either unilateral or bilateral) were considered as having stage I disease (IE for one orbit, and “IE-bilateral” for both orbits involvement). For all other sites, the Ann Arbor staging system was applied [26].

### 2.2. Imaging

The [^18^F]FDG PET-CT studies were performed on PET-CT scanners (GE Healthcare; DISCOVERY 690 and DISCOVERY MI; 7 to 8 frames; frame time 1.5–3 min), according to our standard protocol, with the administration of a diluted oral contrast agent and injection of 3.7 MBq/kg [^18^F]FDG approximately 60 min prior to the study.

For all included studies, we documented whether an extranodal lesion and/or nodal involvement were detected on PET. In each study, the maximum standardized uptake value (SUVmax), metabolic tumor volume (MTV) and total lesion glycolysis (TLG) values were documented in the “hottest” extranodal lesion and in the “hottest” nodal lesion. MTV was obtained with the 41% SUVmax threshold method [27] using Q.Volumetrix AI (GE Healthcare). The TLG value was computed as the product of the measured mean standardized uptake value and MTV.

### 2.3. Outcome Data

Associations between [^18^F]FDG-avidity and staging, laboratory and pathological parameters were assessed. On a survival analysis, the endpoint evaluated was progression-free-survival (PFS), defined as the time from the staging [^18^F]FDG PET-CT study to disease progression that necessitated a change in the primary clinical strategy, or to death from any cause.

### 2.4. Statistical Analysis

Categorical data were described with contingency tables that included frequency and percent. Continuous variables were evaluated for normal distribution and reported as median and interquartile range (IQR). Medians of continuous variables were used as cutoffs for defining dichotomous variables (i.e., values above the median were taken as positive). Pearson’s χ^2^ test and Fisher’s exact test were used to compare rates of categorical variables. The Mann–Whitney U test and the Wilcoxon signed-rank test were used to compare medians of continuous variables between two unpaired and paired groups, respectively. The median length of follow-up was measured using reverse censoring method. The median survival time and the probabilities of PFS were estimated with the Kaplan–Meier method. Log-rank test and univariate Cox regression were applied to study the crude association between the studied predictors and PFS. A multivariate Cox regression analysis was performed using a backward method (*p* > 0.1 was used as a criterion for removal) in order to identify independent predictors for PFS. Variables with a trend or a significant association with PFS, as well as those known to be associated with prognosis (age and serum LDH) were tested in the multivariate analysis. In the presented survival curves, the continuous variable (SUVmax) was dichotomized at the optimal threshold derived from the “maxstat” R package [28]. A two-sided *p* value of <0.05 was considered statistically significant. SPSS software (IBM SPSS Statistics for Windows, version 27, IBM Corp., Armonk, NY, USA, 2017) was used for statistical analysis. Survival curves were generated using the open-source statistics software R (version 4.0.5, R Foundation for Statistical Computing).

## 3. Results

### 3.1. Patient Characteristics

The 66 study patients had a median age of 69.4 years. Thirty-seven (56%) and 29 (44%) patients had stage I and IV disease, respectively. The most common extranodal locations were stomach (28.8%), orbital (22.7%) and subcutaneous-tissue (15.2%). Only a minority of the patients had high Ki-67 index (3.8%), high serum LDH (5.7%) or thrombocytopenia (8.7%). The patients were clinically managed after imaging with different strategies: 27 patients (40.9%) were followed under a WW policy, 19 patients (28.8%) started a systemic therapy, 18 patients (27.3%) were referred to radiotherapy, and 2 patients (3%) were referred to surgical resection. Systemic therapies included immunotherapy (rituximab monotherapy, *n* = 5) and chemoimmunotherapy (*n* = 14). The most frequently employed chemoimmunotherapy protocol was R-CVP (rituximab, cyclophosphamide, vincristine, prednisolone, *n* = 9), followed by BR (bendamustine plus rituximab, *n* = 5) and rituximab plus chlorambucil (*n* = 1). Table 1 summarizes the clinical characteristics of the study patients.

### 3.2. PET Characteristics of Extranodal Lesions

**Figure 1:**
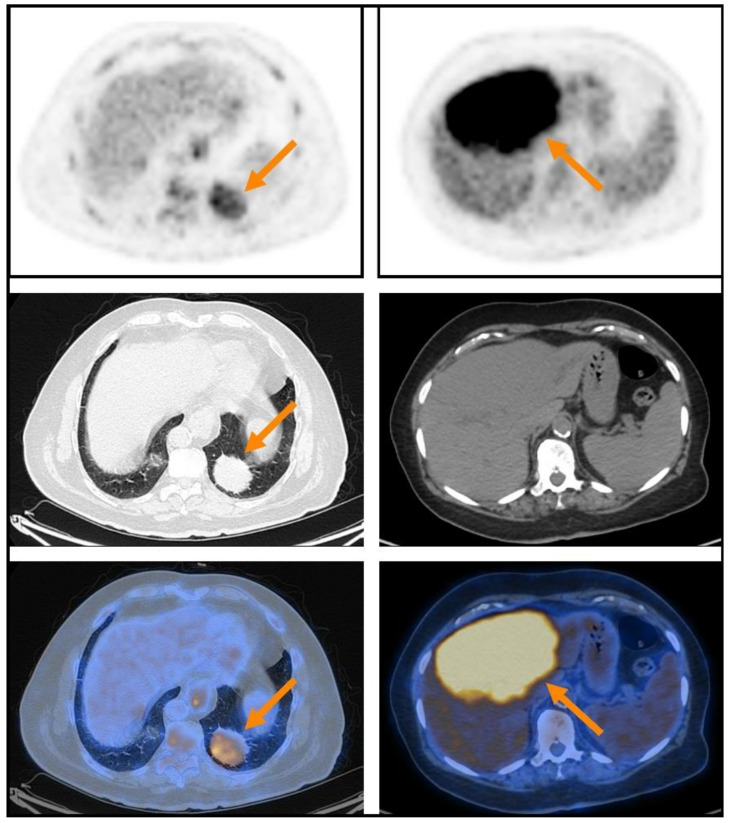
Two illustrative extranodal MALT lymphoma lesions that were detected on PET (upper row), presented together with corresponding CT (middle row) and fusion PET-CT (lower row) transaxial slices: a case of lung MALT lymphoma with lower [^18^F]FDG-avidity (SUVmax 5.9) on the left, and a case of liver MALT lymphoma with higher [^18^F]FDG-avidity (SUVmax 9.0) on the right. Both patients were referred to systemic therapy after imaging. While the patient with the lung lesion did not progress during a follow-up period of 6.4 years, the patient with the liver lesion progressed 2.4 years after staging.

Extranodal lesions were identified on PET in 38/66 (57.6%) [^18^F]FDG PET-CT studies. Table 2 details the detection rates of extranodal lesions in the whole cohort as well as in specific locations. While detection rate was 100% in subcutaneous-tissue, breast, lung and liver lesions, detection rates were much lower in those located in the stomach (26.3%), intestinal tract (28.6%), urinary bladder (50%) and orbit (60%). Detection rate of lesions located in tissues known to have high/heterogeneous physiologic [^18^F]FDG-uptake (gastrointestinal tract, orbit, head and neck, urinary bladder) was significantly lower than that of lesions located in tissues known to have low/homogenous physiologic [^18^F]FDG-uptake (subcutaneous-tissue, breast, lung, liver) (40.4% vs. 100%, *p* < 0.01, Table 3, Figure 1). The extranodal lesions identified on PET imaging had median SUVmax, MTV and TLG of 7.1, 5.5 mL and 25.6 g, respectively (Table 2). These variables were not significantly different in different anatomic locations.

### 3.3. PET Characteristics of Nodal Lesions

Nodal involvement was recorded in 22/66 (33.3%) study patients based on imaging. Thirteen patients (19.7% of the total cohort) had [^18^F]FDG-avid nodal disease identified on PET imaging, and nine patients (13.6% of the total cohort) had non-[^18^F]FDG-avid lymphadenopathy identified on the CT part of the scan, suggesting involvement by lymphoma (e.g., multiple enlarged lymph nodes identified in several nodal stations). Rates of [^18^F]FDG-avid nodal lymphoma were not significantly different among patients with MALT lymphoma of different extranodal locations (Table 2 and Table 3). The nodal lesions identified on PET imaging had median SUVmax, MTV and TLG of 5, 2.1 mL and 7.3 g, respectively (Table 2). Among patients whose PET identified both nodal and extranodal [^18^F]FDG-avid disease, the median SUVmax, MTV and TLG of the “hottest” nodal lesions were significantly lower than those calculated in the matched “hottest” extrandodal lesions (Table 4).

### 3.4. PET Characteristics of Extranodal Lesions Are Associated with Staging Variables

We analyzed the difference between three groups of the total cohort: 28 patients whose extranodal lesions were not detected on PET, 19 patients whose extranodal lesions showed low [^18^F]FDG-avidity (SUVmax < median SUVmax) and 19 patients whose extranodal lesions showed high [^18^F]FDG-avidity on PET. Table 5 details the differences identified between the groups, particularly in variables that reflect disease burden: stage of the lymphoma (*p* = 0.02), rates of bulky disease (*p* < 0.01), BM involvement (*p* = 0.02) and coexisting [^18^F]FDG-positive nodal involvement (*p* = 0.02).

### 3.5. Prediction of PFS

At the time of the analysis, the study patients had a median follow-up of 31.2 (IQR 14.4—74.9) months, and their median PFS was 38.0 (IQR 12.4-110.1) months from their staging [^18^F]FDG PET-CT. 

**Figure 2:**
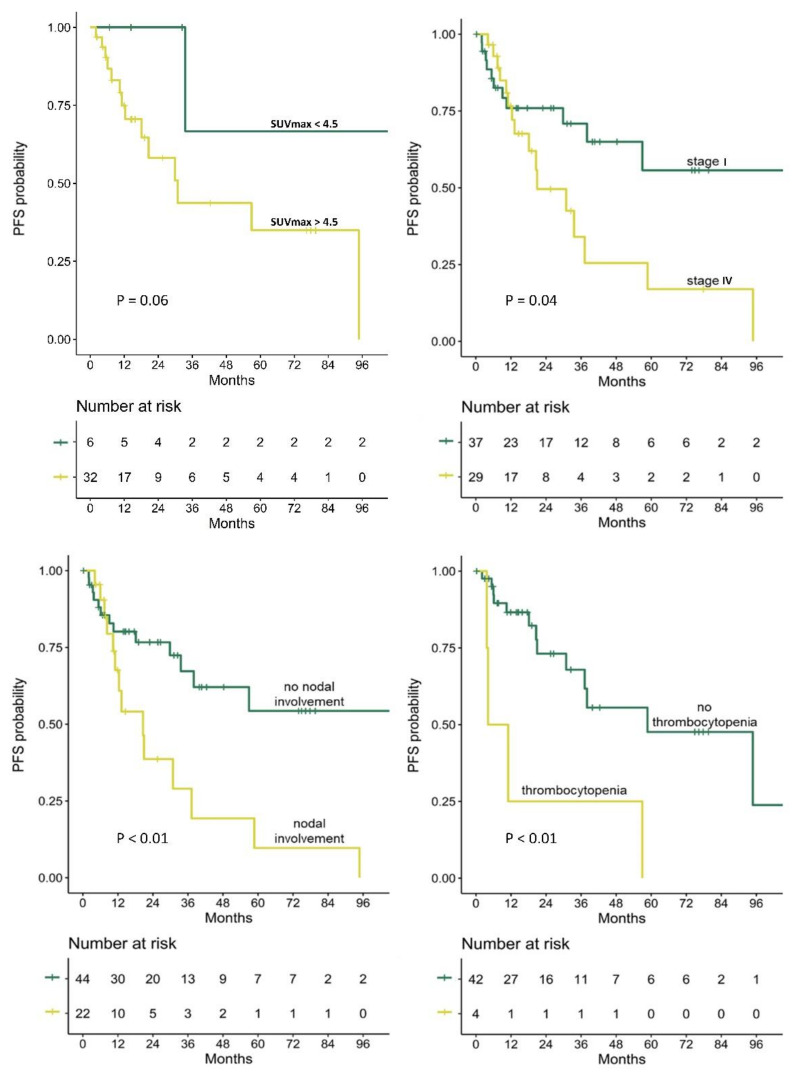
Kaplan–Meier curves of PFS probability in (upper left) patients whose extranodal MALT lymphoma lesions were detected on PET and had SUVmax greater vs lower than 4.5, (upper right) patients with stage I vs IV disease, (lower left) patients with vs without nodal involvement, and (lower right) patients with vs without thrombocytopenia. PFS, progression-free survival; SUVmax, maximum standardized uptake value; Log-rank *p*-value is presented for each survival analysis.

In a Cox regression analysis for PFS that included the entire cohort (shown in Table 6, Figure 2), several variables were found to be significantly predictive of PFS. Disease stage, a concomitant nodal involvement, and thrombocytopenia were found to be associated with shorter PFS (*p* = 0.05, *p* < 0.01 and *p* < 0.01, respectively). Of the analyzed PET parameters, the SUVmax recorded in extranodal lesions identified on PET was associated with shorter PFS (HR 1.10, 95% CI 1.01–1.19, *p* = 0.02), while MTV and TLG of the lesions had no statistically significant impact on PFS (*p* = 0.26 and *p* = 0.11, respectively). In a multivariate Cox analysis, SUVmax and thrombocytopenia were the only significant predictors of PFS (HR 1.24, 95% CI 1.04–1.48, *p* = 0.01, and HR 10.31, 95% CI 1.27–83.50, *p* = 0.03, respectively).

In subgroup analyses performed separately for patients with stage I and stage IV disease (Tables S1 and S2 in the supplementary file, respectively), higher SUVmax and TLG parameters of the extranodal lesion detected on PET showed trends towards shorter PFS in patients with localized disease only (*p* = 0.09 and *p* = 0.10, respectively).

## 4. Discussion

[^18^F]FDG PET-CT imaging has achieved an unsurpassed reputation in the field of lymphomas. However, identifying disease extent of MALT lymphoma on [^18^F]FDG PET-CT poses a particular challenge. Not only the somewhat low [^18^F]FDG-avidity of MALT lymphoma extranodal lesions, verified again in the current study (median SUVmax of 7.1), but their tendency to be located in tissues with physiologic [^18^F]FDG-uptake can make image interpretation difficult. In our data, more than two-thirds of the studied extranodal lesions were located in tissues that have high/heterogenous physiologic [^18^F]FDG-uptake, e.g., along the digestive tract and in proximity to extraocular muscles. In most of such cases we found that the interpreting physician could not confidently identify the extranodal lesion on PET. Such extranodal lesions are not necessarily non-[^18^F]FDG-avid, but their [^18^F]FDG-avidity might not be high enough to overcome the background physiologic [^18^F]FDG-uptake. In contrast, our study highlight the excellent detection (100%) found among extranodal MALT lymphoma lesions located in tissues that have low/homogenous physiologic [^18^F]FDG-uptake, such as subcutaneous-tissue, lungs, and liver. In such cases, [^18^F]FDG PET-CT could be the modality of choice for primary anatomical staging.

[^18^F]FDG-avid nodal disease was detected in one fifth of the study patients. The detection rate of nodal disease on PET, as well as the SUVmax, MTV and TLG calculated in these lesions did not differ significantly in patients with extranodal lesions located in different sites. However, these PET-derived parameters were all significantly lower in nodal lesions compared with matched extranodal lesions. These observations, which were not described in previous studies, possibly reflect that in patients with concomitant nodal and extranodal involvement, extranodal sites may possess different biological characteristics than those nodal sites do.

Our results indeed support the hypothesis that higher glucose metabolism is associated with lymphoma behavior and aggressiveness. We report in the current study an association between [^18^F]FDG-avidity of extranodal lesions and staging variables, including lymphoma’s stage, rates of bulky disease, BM involvement, and [^18^F]FDG-avid nodal involvement, associations that were reported in previous studies [8,9]. However, our results did not show the previously reported association between [^18^F]FDG-avidity and mitotic index [9,29,30], probably due to low number of cases with high Ki-67 index and the lack of such data in some included cases.

In line with that, we also found that the SUVmax measured in involved extranodal lesions was significantly associated with a shorter PFS, suggesting that PET-based parameters may reflect disease aggressiveness and provide prognostic information. However, this result should be interpreted with caution. While it supports previous reported associations between SUVmax and overall-survival [19,21] and between TLG and PFS [22] in MALT lymphoma patients, it contradicts other previous reports. In a study that included 64 MALT lymphoma cases, pretreatment [^18^F]FDG PET-CT findings did not correlate with PFS [20], and in a larger study on 161 cases, SUV, MTV, nor TLG correlated with PFS [23]. Still, the latter studies did not present subanalysis for patients with localized disease, and our results suggest a possible association between PFS and PET-based parameters in such patients.

The present study suffers several limitations. Since the incidence of MALT lymphoma is low and the number of included patients was hence relatively small, we had to include all patients in the PFS analysis, despite their heterogeneity, in order to gain the statistical power to find significant predictors. Therefore, and in light of contradicting results on such a debated topic, larger studies or subgroup-specific studies are warranted to better define the prognostic role of [^18^F]FDG PET-CT in patients with MALT lymphoma.

## 5. Conclusions

Detection rate of extranodal MALT lymphoma lesions located in tissues with high/heterogeneous physiologic [^18^F]FDG-uptake (e.g., along the digestivhe tract) appears to be low on [^18^F]FDG PET-CT. However, detection rate of extranodal MALT lymphoma lesions located in other tissues (e.g., subcutaneous-tissues, lung and liver) is excellent, and [^18^F]FDG PET-CT should be recommended for staging such cases. Our results indicate that PET-based parameters measured in detected extranodal MALT lymphoma lesions may be associated with PFS, although verification of this observation in larger studies is still required.

## Figures and Tables

**Table 1:** Patient characteristics.

	Variable	Value
	Age (years)	69.4 (57.7–74.6)
	Female	39/66 (59.1%)
Extranodal location	Stomach	19/66 (28.8%)
	Orbital	15/66 (22.7%)
	Subcutaneous-tissue (including breast)	10/66 (15.2%)
	Gastrointestinal tract (excluding stomach)	7/66 (10.6%)
	Lung	6/66 (9.1%)
	Head and Neck	4/66 (6.1%)
	Liver	2/66 (3%)
	Other locations	3/66 (4.5%)
Clinical staging data	Stage I	37/66 (56%)
	Stage IV	29/66 (44%)
	B symptoms	6/58 (10.3%)
	Bulky disease	11/66 (16.7%)
	BM involvement	8/45 (17.8%)
	Nodal involvement	22/66 (33.3%)
Pathology and laboratory data	High Ki-67 index	2/53 (3.8%)
	High serum LDH	3/57 (5.3%)
	Anemia	14/48 (29.2%)
	Thrombocytopenia	4/46 (8.7%)
PET data	Detection of extranodal disease	38/66 (57.6%)
	Detection of nodal disease	13/66 (19.7%)
Primary clinical strategy	Watch-and-wait strategy	27/66 (40.9%)
	Systemic therapy	19/66 (28.8%)
	Radiotherapy	18/66 (27.3%)
	Surgical resection	2/66 (3%)

Categorical variables are reported as frequency and percentage. Continuous variables are reported as median and IQR. Head and neck cases included extranodal lesions located in the pharynx, thyroid or parotid gland; Bulky disease, positive if any lymphoma lesion > than 5 cm in any dimension; Nodal involvement, positive if any lymph node was considered involved in the disease (not necessarily [^18^F]FDG-avid); BM involvement, positive if lymphoma was identified on BM aspirate or biopsy or flow cytometric immunophenotyping; High Ki-67 index, positive if reported >20%; High serum LDH, positive if >380 U/l; Anemia, positive if hemoglobin <12.5 g/dL for female or <13.5 g/dL for male patient; Thrombocytopenia, positive if platelets count <150,000 platelets per microliter; BM, bone marrow; LDH, lactate dehydrogenase.

**Table 2:** PET characteristics of extranodal and nodal lesions.

		All Studies (*n* = 66)	Gastric (*n* = 19)	Orbital (*n* = 15)	Subcutaneous-Tissue (*n* = 10)	GI (exc. Stomach) (*n* = 7)	Lung (*n* = 6)	Head and Neck (*n* = 4)	Liver (*n* = 2)
Extranodal lesion:	DR	38 (57.6%)	5 (26.3%)	9 (60%)	10 (100%)	2 (28.6%)	6 (100%)	2 (50%)	2 (100%)
	SUVmax	7.166 (4.8–9.3)	8.3 (4.8–9.9)	7.6 (5.1–8.6)	5.5 (3.2–8.8)	8.2	6.4 (4.1–9.5)	20.3	8.0
	MTV	5.5 (3.2–22.5)	12.1 (4.4–221)	4.5 (3.2–6.5)	9.8 (4.8–38.2)	222.1	9.8 (4.7–19.4)	70.0	601.8
	TLG	25.6 (11.4–100.7)	57.8 (13.8–1308)	22.8 (12.6–34.6)	35.7 (4.8–146)	1342.4	37.1 (17–100.7)	1580.7	3488.5
Nodallesion:	DR	13 (19.7%)	3 (15.8%)	3 (20%)	2 (20%)	3 (42.9%)	1 (16.7%)	0 (0%)	0 (0%)
	SUVmax	5 (4.4–10.5)	7.7	4.7	6.2	4.4	5.0	-	-
	MTV	2.1 (1.7–9.3)	10.8	1.7	1.1	2.4	2.1	-	-
	TLG	7.3 (5.3–81.6)	124	8.3	4.1	6.4	7.3	-	-

Categorical variables are reported as frequency and percentage. Continuous variables are reported as median and IQR if *n* > 3 and as median if *n* ≤ 3. SUVmax, MTV and TLG were calculated in the “hottest” extranodal and “hottest” nodal lesions, only when detected on PET. DR, detection rate; SUVmax, maximum standardized uptake value; MTV, metabolic tumor volume; TLG, total lesion glycolysis.

**Table 3:** PET characteristics based on the physiologic [^18^F]FDG-uptake in the location of the extranodal lesion.

Caption		All Studies	Studies with Extranodal Lesion Located in Tissues with High/Heterogeneous Physiologic [^18^F]FDG-Uptake	Studies with Extranodal Lesion Located in Tissues with Low/Homogeneous Physiologic [^18^F]FDG-Uptake	*p*
Extranodal lesion	DR:	38/66 (57.6%)	19/47 (40.4%)	19/19 (100%)	<0.01
	SUVmax	7.1 (4.8–9.3)	7.9 (4.9–11.2)	6.8 (4.3–9.1)	0.25
	MTV	5.5 (3.2–22.5)	5.4 (3.0–12.1)	11.7 (3.6–28)	0.40
	TLG	25.6 (11.4–100.7)	22.8 (10.4–57.8)	47.9 (11.7–112.2)	0.71
Nodal lesion	DR:	13/66 (19.7%)	9/47 (19.9%)	4/19 (21.1%)	0.86

Categorical variable is reported as frequency and percentage. Continuous variables are reported as median and IQR. High/heterogeneous physiologic [^18^F]FDG-uptake, refers to gastrointestinal tract, orbit, head and neck, urinary bladder. Low/homogenous physiologic [^18^F]FDG-uptake, refers to subcutaneous-tissue, breast, lung, liver. SUVmax, MTV and TLG were calculated in the “hottest” extranodal and “hottest” nodal lesions, only if identified on PET. DR, detection rate; SUVmax, maximum standardized uptake value; MTV, metabolic tumor volume; TLG, total lesion glycolysis.

**Table 4:** Comparison between extranodal and nodal lesions.

Caption	Extranodal Lesion	Nodal Lesion	*p*
SUVmax	10.5 (7.7–11.3)	5.0 (4.4–9.4)	0.04
MTV	7.7 (3.9–157.6)	2.0 (1.5–11.6)	0.02
TLG	49.5 (22.7–959.8)	7.8 (4.7–75.2)	0.02

Continuous variables are reported as median and IQR. This comparison included studies that identified both extranodal and nodal lesions (*n* = 10). PET parameters of the “hottest” extranodal lesion and the “hottest” nodal lesion were compared. SUVmax, maximum standardized uptake value; MTV, metabolic tumor volume; TLG, total lesion glycolysis.

**Table 5:** Comparison of patients’ characteristics based on extranodal lesion’s PET characteristics.

Variable	Extranodal Lesion Not Detected on PET (*n* = 28)	Extranodal Lesion with Low [^18^F]FDG-Avidity (*n* = 19)	Extranodal Lesion with High [^18^F]FDG-Avidity (*n* = 19)	*p*
Stage I	21/28 (75%)	9/19 (47.4%)	7/19 (36.8%)	0.02 ^(a)^
Stage IV	7/28 (25%)	10/19 (52.6%)	12/19 (63.2%)	0.02 ^(a)^
B symptoms	3/24 (12.5%)	0/17 (0%)	3/17 (17.6%)	0.10
Bulky disease	0/28 (0%)	5/19 (26.3%)	6/19 (31.6%)	<0.01 ^(a)(b)^
BM involvement	1/20 (5%)	1/11 (9.1%)	6/14 (42.9%)	0.02 ^(a)^
Nodal involvement	7/28 (25%)	6/19 (31.6%)	9/19 (47.4%)	0.28
High Ki-67 index	1/21 (4.8%)	0/15 (0%)	1/17 (5.9%)	0.50
High serum LDH	0/25 (0%)	3/16 (18.8%)	0/16 (0%)	0.02 ^(b)^
Anemia	6/18 (33.3%)	4/15 (26.7%)	4/15 (26.7%)	0.88
Thrombocytopenia	1/18 (5.6%)	1/13 (7.7%)	2/15 (13.3%)	0.73
[^18^F]FDG-avid nodal disease	3/28 (10.7%)	2/19 (10.5%)	8/19 (42.1%)	0.02 ^(c)^

Categorical variables are reported as frequency and percentage. Continuous variables are reported as median and IQR. [^18^F]FDG-avidity was considered high if SUVmax was > median SUVmax. Bulky disease, positive if any lymphoma lesion >5 cm in any dimension; Nodal involvement, positive if any lymph node was considered involved in the disease (not necessarily [^18^F]FDG-avid); BM involvement, positive if lymphoma was identified on BM aspirate or biopsy or flow cytometric immunophenotyping; High Ki-67 index, positive if reported >20%; High serum LDH, positive if >380 U/l; Anemia, positive if hemoglobin <12.5 g/dL for female or <13.5 g/dL for male patient; Thrombocytopenia, positive if platelets count <150,000 platelets per microliter; BM, bone marrow; LDH, lactate dehydrogenase; ^(a)^, significant difference between the 1st and 3rd column; ^(b)^, significant difference between the 1st and 2nd column; ^(c)^, significant difference between the 2nd and 3rd column.

**Table 6:** Cox analysis for PFS.

Variable	Variable	*p*	HR (95% CI)
Clinical characteristics	Age	0.37	1.01 (0.98–1.05)
	Female	0.48	1.32 (0.61–2.83)
	Stage I (vs stage IV)	0.05 *	0.45 (0.21–0.98)
	B symptoms	0.10	2.52 (0.84–7.62)
	Bulky disease	0.69	0.78 (0.23–2.62)
	BM involvement	0.44	1.51 (0.54–4.20)
	Nodal involvement	<0.01 *	2.90 (1.34–6.24)
	High Ki-67 index	0.14	5.09 (0.59–43.85)
	High serum LDH	0.50	0.05 (0.01–399.32)
	Anemia	0.53	1.35 (0.52–3.50)
	Thrombocytopenia	<0.01 *^	4.85 (1.53–15.40)
PET characteristics	Extranodal lesion detected (vs not detected) on PET	0.77	0.89 (0.41–1.93)
	SUVmax of detected extranodal lesions	0.02 *^	1.10 (1.01–1.19)
	MTV of detected extranodal lesions	0.26	1.00 (1.00–1.00)
	TLG of detected extranodal lesions	0.11	1.00 (1.00–1.00)
	Nodal disease detected (vs not detected) on PET	0.21	1.82 (0.72–4.63)
	SUVmax of detected nodal disease	0.67	1.03 (0.91–1.16)
	MTV of detected nodal disease	0.56	1.02 (0.95–1.11)
	TLG of detected nodal disease	0.71	1.00 (0.99–1.02)

In a univariable Cox regression for PFS, the four variables marked with * were identified as predictors of PFS. Hazard ratios (HR) with 95% confidence intervals (CI) are presented for all analyzed variables. Variables marked with ^ were further verified in a multivariate analysis. Bulky disease, positive if any lymphoma lesion >5 cm in any dimension; Nodal involvement, positive if any lymph node was considered involved in the disease (not necessarily [^18^F]FDG-avid); BM involvement, positive if lymphoma was identified on BM aspirate or biopsy or flow cytometric immunophenotyping; High Ki-67 index, positive if reported >20%; High serum LDH, positive if >380 U/l; Anemia, positive if hemoglobin <12.5 g/dL for female or <13.5 g/dL for male patient; Thrombocytopenia, positive if platelets count <150,000 platelets per microliter; BM, bone marrow; LDH, lactate dehydrogenase; SUVmax, maximum standardized uptake value; MTV, metabolic tumor volume; TLG, total lesion glycolysis; SUVmax, MTV, and TLG were analyzed as continuous variables.

## Data Availability

The datasets used and/or analyzed during the current study are available from the corresponding author on reasonable request.

## Supplementary Materials

The following are available online at https://www.mdpi.com/article/10.3390/cancers14030750/s1, Table S1: Univariable analysis for PFS in patients with stage I disease, Table S2: Univariable analysis for PFS in patients with stage IV disease.
