# Supplementary material for: Is There a Role for [18F]FDG PET-CT in Staging MALT Lymphoma?
Source: Cancers (Basel). 2022 Jan 31;14(3):750. doi: 10.3390/cancers14030750 (PMC8833535; doi:10.3390/cancers14030750)
Supplement: Supplementary file 1 [file cancers-14-00750-s001.zip › cancers-1511299-supplementary.pdf]

cancers

Table S1. Cox analysis for PFS in patients with stage I disease.

Variable	Variable	P	HR (95% CI)
Clinical characteristics	Age	0.85	1.00 (0.96–1.05)
	Female	0.77	1.19 (0.38–3.75)
	B symptoms	0.53	1.97 (0.24–16.15)
	Bulky disease	0.27	2.44 (0.50–11.83)
	BM involvement	0.32	2.94 (0.35–25.05)
	Anemia	0.51	1.73 (0.35–8.65)
	Thrombocytopenia	0.11	3.97 (0.73–21.44)
PET characteristics	Extranodal lesion detected (vs not detected) on PET	0.96	0.97 (0.29–3.25)
	SUVmax of detected extranodal lesion	0.09	1.08 (0.99–1.17)
	MTV of detected extranodal lesion	0.28	1.00 (1.00–1.01)
	TLG of detected extranodal lesion	0.12	1.00 (1.00–1.00)
	TLG of extranodal lesion > median TLG	0.10	2.81 (0.82–9.67)

Univariable Cox regression for PFS. Hazard ratios (HR) with 95% confidence intervals (CI) are presented for all analyzed variables. Bulky disease, positive if any lymphoma lesion > 5 cm in any dimension; BM involvement, positive if lymphoma was identified on BM aspirate or biopsy or flow cytometric immunophenotyping; Anemia, positive if hemoglobin < 12.5 g/dL for female or < 13.5 g/dL for male patient; Thrombocytopenia, positive if platelets count < 150,000 platelets per microliter; BM, bone marrow; SUVmax, maximum standardized uptake value; MTV, metabolic tumor volume; TLG, total lesion glycolysis.

Table S2. Cox analysis for PFS in patients with stage IV disease.

Variable	Variable	P	HR (95% CI)
Clinical characteristics	Age	0.25	1.03 (0.98–1.08)
	Female	0.47	1.49 (0.50–4.43)
	B symptoms	0.07	4.15 (0.91–18.99)
	Bulky disease	0.12	0.20 (0.03–1.55)
	BM involvement	0.79	0.84 (0.22–3.16)
	Nodal involvement	0.11	3.44 (0.77–15.34)
	High Ki-67 index	0.06	15.54 (0.94–256.39)
	High serum LDH	0.44	0.04 (0–154.70)
	Anemia	0.89	0.92 (0.26–3.27)
	Thrombocytopenia	0.01*	27.48 (2.46–307.73)
PET characteristics	Extranodal lesion detected (vs not detected) on PET	0.31	1.72 (0.60–5.00)
	SUVmax of detected extranodal lesion	0.81	1.03 (0.83–1.26)
	MTV of detected extranodal lesion	0.53	1.00 (1.00–1.01)
	TLG of detected extranodal lesion	0.60	1.00 (1.00–1.00)
	Nodal disease detected (vs not detected) on PET	0.44	0.66 (0.23–1.89)
	SUVmax of detected nodal disease	0.57	1.14 (0.73–1.78)
	MTV of detected nodal disease	0.50	1.04 (0.93–1.17)
	TLG of detected nodal disease	0.34	1.01 (0.99–1.04)

Univariable Cox regression for PFS. Hazard ratios (HR) with 95% confidence intervals (CI) are presented for all analyzed variables. Bulky disease, positive if any lymphoma lesion > 5 cm in any dimension; Nodal involvement, positive if any lymph node was considered involved in the disease (not necessarily [^{18}F]FDG-avid); BM involvement, positive if lymphoma was identified on BM aspirate or biopsy or flow cytometric immunophenotyping; High Ki-67 index, positive if reported > 20%; High serum LDH, positive if > 380 U/l; Anemia, positive if hemoglobin < 12.5 g/dL for female or < 13.5 g/dL for male patient; Thrombocytopenia, positive if platelets count < 150,000 platelets per microliter; BM, bone marrow; LDH, lactate dehydrogenase; SUVmax, maximum standardized uptake value; MTV, metabolic tumor volume; TLG, total lesion glycolysis.
